# Ocular dryness in intensive care: proposal for a new nursing diagnosis

**DOI:** 10.1590/0034-7167-2022-0698

**Published:** 2023-11-13

**Authors:** Jéssica Naiara de Medeiros Araújo, Ana Paula Nunes de Lima Fernandes, Ana Clara Dantas, Marcos Antonio Ferreira, Marcos Venícios de Oliveira Lopes, Allyne Fortes Vitor

**Affiliations:** I Universidade do Estado do Rio Grande do Norte. Caicó, Rio Grande do Norte, Brazil; II Faculdade de Enfermagem e de Medicina Nova Esperança. Mossoró, Rio Grande do Norte, Brazil; III Universidade Federal do Rio Grande do Norte. Natal, Rio Grande do Norte, Brazil; IV Universidade Federal de Mato Grosso do Sul. Campo Grande, Mato Grosso do Sul, Brazil; V Universidade Federal do Ceará. Fortaleza, Ceará, Brazil

**Keywords:** Nursing Diagnosis, Concept Formation, Desiccation, Eye, Intensive Care Units, Diagnóstico de Enfermería, Formación de Concepto, Desecación, Ojo, Unidades de Cuidados Intensivos, Diagnóstico de Enfermagem, Formação de Conceito, Ressecamento, Olho, Unidades de Terapia Intensiva

## Abstract

**Objective:**

to analyze the concept associated with diagnostic proposition Ocular dryness in adult patients hospitalized in an Intensive Care Unit, identifying its attributes, antecedents and consequences.

**Methods:**

a methodological study carried out through concept analysis, operationalized by scoping review.

**Results:**

the analysis of 180 studies allowed the identification of two attributes, 32 antecedents and 12 consequences. The attributes were tear film deficiency and ocular signs and/or symptoms. The prevalent antecedents were incomplete eyelid closure (lagophthalmos) and blinking mechanism decrease. Major consequences included conjunctival hyperemia and decreased tear volume.

**Conclusions:**

this study allowed constructing nursing diagnosis Ocular dryness, part of domain 11, class 2, with 12 defining characteristics, 12 related factors, seven populations at risk and 13 associated conditions. This problem-focused proposal may provide targeted care by promoting early detection and implementing interventions that reduce the risk of ocular damage.

## RESUMO

**Objetivo:**

analisar o conceito associado à proposição do diagnóstico de enfermagem Ressecamento ocular em pacientes adultos internados em Unidade de Terapia Intensiva com identificação de seus atributos, antecedentes e consequentes.

**Métodos:**

estudo metodológico, realizado mediante análise de conceito operacionalizada por *scoping review*.

**Resultados:**

a análise dos 180 estudos permitiu identificação de dois atributos, 32 antecedentes e 12 consequentes. Os atributos foram deficiência do filme lacrimal e sinais e/ ou sintomas oculares. Os antecedentes prevalentes foram fechamento palpebral incompleto (lagoftalmia) e mecanismo de piscar diminuído. Os principais consequentes incluíram hiperemia conjuntival e volumetria lacrimal diminuída.

**Conclusões:**

este estudo permitiu a construção do diagnóstico de enfermagem Ressecamento ocular, integrante do domínio 11, classe 2, com 12 características definidoras, 12 fatores relacionados, sete populações em risco e 13 condições associadas. Essa proposta com foco no problema poderá proporcionar cuidado direcionado, promovendo detecção precoce e execução de intervenções que reduzam o risco de danos oculares.

## RESUMEN

**Objetivo:**

analizar el concepto asociado a la proposición del diagnóstico de enfermería Sequedad ocular en pacientes adultos internados en una unidad de cuidados intensivos, identificando sus atributos, antecedentes y consecuencias.

**Métodos:**

estudio metodológico, realizado a través del análisis de concepto operacionalizado por scoping review.

**Resultados:**

el análisis de 180 estudios permitió identificar dos atributos, 32 antecedentes y 12 consecuentes. Los atributos fueron deficiencia de película lagrimal y signos y/o síntomas oculares. Los antecedentes prevalentes fueron cierre palpebral incompleto (lagoftalmos) y disminución del mecanismo de parpadeo. Las principales consecuencias incluyeron hiperemia conjuntival y disminución del volumen lagrimal.

**Conclusiones:**

este estudio permitió la construcción del diagnóstico de enfermería Sequedad ocular, parte del dominio 11, clase 2, con 12 características definidoras, 12 factores relacionados, siete poblaciones de riesgo y 13 condiciones asociadas. Esta propuesta centrada en el problema puede brindar atención dirigida al promover la detección temprana e implementar intervenciones que reduzcan el riesgo de daño ocular.

## INTRODUCTION

Patients hospitalized in the Intensive Care Unit (ICU) are more likely to manifest changes in the ocular surface because most of them are in a serious general condition. Thus, they generally require complex care, ventilatory assistance, invasive procedures and the use of various medications, such as sedatives and neuromuscular blockers to maintain clinical therapy and vital parameters, which can lead to ocular changes. In this way, the importance of eye care is emphasized in order to avoid complications on the ocular surface until the possible evolution to vision loss^(^ 1,2 ^)^.

When relating ocular alterations and length of stay of patients hospitalized in the ICU, a previous study carried out with 230 patients hospitalized in the ICU found an incidence of 53.0% of dry eye in the assessed participants, with an average time of onset of 3.5 days^(^ 3 ^)^. Research carried out with 130 patients observed an incidence of 25.8% of dry eye and corneal abrasion, with an average time of onset of 4 days after admission to the ICU^(^ 4 ^)^. Another study carried out with patients on mechanical ventilation treated with neuromuscular blockade observed a 26.7% incidence of dry eye^(^ 5 ^)^.

The health team must provide a safe environment, promote early detection of the problem, and perform interventions that reduce the risk of eye damage. In this context, nursing plays an essential role by providing direct assistance to patients in order to avoid serious injuries resulting from ocular dryness and maintain cornea integrity. However, care is often postponed due to the lack of knowledge of nurses and the multidisciplinary team about the eye anatomy and physiology, ocular assessment and care that can be implemented to avoid possible damage^(^ 2,6 ^)^.

Dry eye is a medical diagnostic term included in the International Statistical Classification of Diseases and Related Health Problems used to refer to various conditions and diseases caused by inadequate eye moisture and lubrication. It is characterized as a loss of tear film homeostasis triggered by the dryness of the ocular surface, evidenced by ocular signs and symptoms such as burning, foreign body sensation, photophobia, hyperemia and visual acuity disorders. Due to team film instability and hyperos-molarity, inflammation and damage to the ocular surface and neurosensory abnormalities play etiological roles^(^ 2,7 ^)^.

In the meantime, it is worth noting that the 2018-2020 version of NANDA-International (NANDA-I) contains nursing diagnosis Risk for ocular dryness (00219), previously named Risk for dry eye, defined as susceptibility to ocular discomfort or damage to the cornea and conjunctiva caused by quantitative and/or qualitative tear film deficiency, responsible for eye moisture that can compromise health^(^ 8 ^)^.

However, the 2018-2020 version of NANDA-I does not have specific nursing diagnoses focusing on the problem of ocular dryness. The International Classification for Nursing Practice (ICNP®) terminology in its 2019-2020 version also does not have diagnoses aimed at ocular dryness^(^ 9 ^)^. In this context, it is essential to unify and delimit the use of a term specifically directed towards nursing with a focus on the problem in understanding an undesirable human response, addressing human responses that exceed the limit of isolated disease conception. Thus, constant research is needed to update the scientific evidence that makes up the systems, in order to standardize the language of diagnoses from a nursing perspective^(^ 8,9,10,11 ^)^.

In the sense of professional appropriation and taking into account this already mentioned context, the present study is justified by the need to use the term “ocular dryness” as an undesirable human response, permeated by an early stage of tear film dysfunction. Notably to this human response, nurses are able to assess its presence, severity and implement specific activities aimed at preventing/treating ocular dryness, using their classification systems^(^ 6,8 ^)^.

Faced with this problem, this study has the following research question: what attributes, antecedents and consequences underlie diagnostic proposition Ocular dryness in adult patients hospitalized in an ICU?

## OBJECTIVE

To analyze the concept associated with diagnostic proposition Ocular dryness in adult patients hospitalized in an ICU, identifying its attributes, antecedents and consequences.

## METHODS

### Ethical aspects

As this is a study that used public domain data, submission to the Research Ethics Committee was not required.

### Study design

This is a methodological study of nursing diagnosis validity, developed using the model proposed by Lopes, Silva and Araú-jo^(^ 12 ^)^, carried out through a concept analysis based on the Walker and Avant model^(^ 13 ^)^, operationalized through scoping review^(^ 14 ^)^.

The concept analysis is the first step to be carried out for the validity process of nursing diagnoses. Identifying attributes, antecedents and consequences of a nursing diagnosis is the purpose of the concept analysis for this study, which are used to identify the definition of diagnosis (attributes), related factors (antecedent) and defining characteristics (consequences). Moreover, at this stage, populations at risk and associated conditions that permeate the diagnosis are also identified^(^ 12 ^)^.

Walker and Avant’s concept analysis model is based on Wilson’s proposal and includes the execution of eight steps, namely: (1) Concept selection; (2) Determination of the objectives of the conceptual analysis; (3) Identification of possible uses of the concept; (4) Determination of critical or essential attributes; (5) Construction of a model case; (6) Development of other cases: borderline, related, invented and illegitimate contrary; (7) Identification of antecedents and consequences of the concept; (8) Definition of empirical references^(^ 13 ^)^. In order to reach the proposed objective, the eight recommended steps were carried out, adapted for the purpose of the diagnostic proposal.

Thus, the concept “Ocular dryness” was chosen in order to analyze it as an undesirable human response in the context of nursing. Regarding the determination of the objectives of the conceptual analysis, this step refers to the purpose of a conceptual analysis that is intended to be carried out^(^ 13 ^)^. The analysis aimed to define the concept of ocular dryness and identify which attributes define ocular dryness and identify its antecedents and consequences.

To determine the critical attributes and identify the antecedents and consequences of the concept, a scoping review was carried out according to the JBI 2020 recommendations^(^ 14 ^)^ and using the criteria established in Preferred Reporting Items for Systematic Reviews and Meta-Analyses extension for Scoping Reviews (PRISMA-ScR)^(^ 15 ^)^. The study in question was registered on the Open Science Framework study platform and a corresponding Uniform Resource Locator sequential identifier (osf.io/qtv79) was generated^(^ 16 ^)^.

### Study protocol/inclusion and exclusion criteria

The scoping review was selected in this study because it allows clarifying key concepts or definitions in the literature and identifying characteristics related to a given concept, including those related to methodological research^(^ 17 ^)^. The review was guided by a research protocol and consisted of the following steps: research question identification; identification of relevant studies; study selection; mapping and extracting the results; and narrative synthesis of results^(^ 14 ^)^.

The Population, Concept and Context (PCC) strategy - P (population), C (concept) and C (context) was applied to guide the research question elaboration. Thus, the selected population consisted of adult patients; the concept was ocular dryness; and the context is related to the ICU. Thus, the main guiding question elaborated was: what is the concept of ocular dryness in adult patients admitted to the ICU? Furthermore, to contemplate the objective of the research for diagnostic construction, the following subsequent research questions were elaborated: what are the attributes that define ocular dryness? What are the antecedents and consequences of ocular dryness?

The search was carried out in November 2018 and updated in August 2022, divided into three parts, in order to identify published and unpublished primary studies (grey literature) as well as reviews^(^ 14 ^)^. The first part of the search took place in the following databases: Scopus (Elsevier), Web of Science and Science Direct.

The following indexed descriptors (Descriptors in Health Sciences - DeCS and Medical Subject Headings - MeSH Database) were delimited according to each database: 1# (Dry Eye Syndromes; *Síndromes de Ojo Seco; Síndromes do Olho Seco*); 2# (Keratocon-juntivite Sicca; *Queratoconjuntivite Seca; Ceratoconjuntivite*); 3# (Dryness; *Sequedad; Ressecamento*); 4# (Intensive Care Units; *Unidades de Cuidados Intensivos; Unidades de Terapia Intensiva*); 5# (Eye; *Ojo; Olho*); and 6# (Retinopathy of Prematurity; *Retinopatía de la Prematuridad; Retinopatia da Prematuridad*).

The crossings in the databases were performed using Boolean operators AND and AND NOT, namely: 1# AND 2# AND 3#; 1# AND 4#; 2# AND 4#; 3# AND 4#; 3# AND 5# and 4# AND 5# AND NOT 6#. Thus, the defined search strategies were: Dry Eye Syndromes AND Keratoconjuntivite Sicca AND Dryness; Dry Eye Syndromes AND Intensive Care Units; Keratoconjuntivite Sicca AND Intensive Care Units; Dryness AND Intensive Care Units; Dryness AND Eye; Intensive Care Units AND Eye AND NOT Retinopathy of Prematurity. In each database, a standardized search was used according to the available strategies. It should be noted that an advanced search was used in each database.

The second part of the search took place in Google Scholar®> using the index and synonym terms (keywords) identified in the studies of the first part of the search: “*Olho seco*”; “Dry eye”; “*Ressecamento ocular*”; “Ocular dryness”; “*Unidade de terapia intensiva*”; “*Intensive care unit*”; “*Cuidados críticos*”; and “Critical care”. The eight strategies used in Google Scholar® for search were: “*olho seco*” and “*unidade de terapia intensiva*” , “ *olho seco*” and “*cuidados críticos*”, “dry eye” and “intensive care unit”, “dry eye” and “critical care”, “*ressecamento ocular*” and “*unidade de terapia intensiva*”, “ *ressecamento ocular*” and “*cuidados críticos*”, “ocular dryness” and “intensive care unit”, “ocular dryness” and “critical care”. Quotation marks were used as a resource in order to limit studies that used the compound term. This stage included the search in gray literature.

For study selection, the following inclusion criteria were used: complete studies available in the databases used and that addressed dry eye and/or presented some element (attributes, antecedents and consequences) of ocular dryness in adult patients in Portuguese, English, Spanish or French. Editorials, letters to the editor, abstracts and articles published more than 25 years ago were excluded from the research, whose time frame was used due to the conceptual changes that occurred about the phenomenon.

The third part of the search took place through a reverse search according to the references identified in the previously selected searches. This scoping review three-part search strategy allows identifying other materials or research not identified in the databases^(^ 14 ^)^.

### Data organization and analysis

The search was carried out in pairs, on different computers, at the same time and using the same internet network. At the end of each part of the search, the results were evaluated through an initial stage of screening the studies, by dynamic reading of titles and abstracts and a subsequent reading of full text by a pair of reviewers, independently. Duplicates were counted only once, and those that did not fit the established eligibility criteria were excluded, and when there were disagreements between reviewers, these were resolved by consensus.

Mapping and data extraction were performed using an instrument designed with the following items: publication identification (identification number, indexed database, study title, file type, authors, country, language, year of publication); methodological aspects (research objective/question, methodology used, type of approach (quantitative/qualitative), level of evidence, population and sample, main findings of the study); and aspects related to concept analysis, construction of diagnosis and definitions (concept defined by the authors, attributes/ characteristics of ocular dryness, antecedents, consequences, conceptual definition of consequences/defining characteristics, empirical references (measurement of diagnosis consequences/ defining characteristics); and operational definition, population at risk and associated conditions). To present the results, tables and charts were used.

The level of evidence of the studies selected in the review was assessed by the Oxford Center Evidence-Based Medicine^(^ 18 ^)^, whose classification is based on: 1a (systematic review of randomized clinical trials); 1b (randomized controlled clinical trial with narrow confidence intervals); 1c (all-or-nothing therapeutic outcomes); 2a (systematic review of cohort studies); 2b (cohort study); 2c (observation of therapeutic results; ecological study); 3a (systematic review of case-control studies); 3b (case-control study); 4 (case reports); and 5 (expert opinion).

After constructing the diagnostic proposition for the NANDA-I taxonomy, Nursing Outcomes Classification (NOC) outcomes and Nursing Interventions Classification (NIC) interventions were suggested, whose selection was based on the researchers’ expertise to support the submission of this proposal and complement the use of standardized languages.

It is worth mentioning that this study was extracted from the doctoral thesis “*Construção e validação do diagnóstico de enfermagem ressecamento ocular em pacientes adultos internados em unidade de terapia intensiva*”, presented to the Health Sciences Center, *Universidade Federal do Rio Grande do Norte*, Natal, RN, Brazil. Available at the UFRN Institutional Repository: https://repositorio.ufrn.br/handle/123456789/26803

## RESULTS

**Figure 1:**
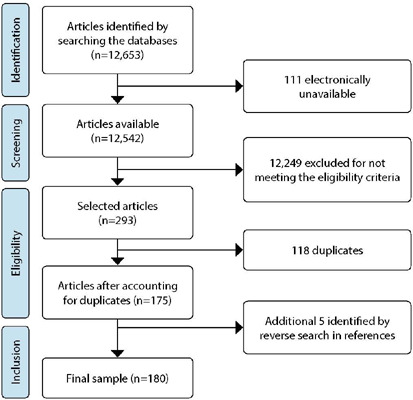
Literature search flowchart and article selection according to the Preferred Reporting Items for Systematic Reviews and Meta-Analyses extension for Scoping Review guidelines, 2022

Searches in the Scopus database (Elsevier) found 2,351 studies, in the Web of Science, 1,943 studies and in Science Direct, 3,422 studies. In Google Scholar®, 4,937 studies were found, totaling 12,653. After applying the eligibility criteria and accounting for duplicates, 175 were selected. Five studies were also included in the reverse search of the reference list, totaling a final sample of 180 studies. Figure 1 shows the search flowchart in the databases and study selection.

According to identified data, in relation to year of publication, the articles dated from 1998 to 2022 and, of these, 19.4% were published in the last five years. As for data source, the majority (42.7%) of studies were identified in Science Direct. The most frequent type of file was an article (95.5%) and, regarding the method of studies, reviews prevailed in 43.3% of the searches. Regarding the level of evidence, level 2c was the most frequent in the sample (33.3%). The most frequent approach was quantitative (66.1%), the continent was America (49.1%), and the language was English in 84.4% of the sample.

The analyzes of the articles that composed the sample allowed identifying diagnostic proposition Ocular dryness components in the general population and in the population admitted to the ICU permeated by the two attributes, 32 antecedents and 12 consequences of the diagnostic focus in question. It is noteworthy that the information was categorized in the following tables by similarity of terms.

The two attributes identified were tear film deficiency in 109 (60.5%) studies and ocular signs and/or symptoms in 73 (40.5%) of studies. The summarization of the two essential attributes identified for Ocular dryness allowed the composition of the diagnostic proposition concept definition.

A total of 32 antecedents were identified, which are described in Table 1, subdivided into individual factors and environmental factors and represented, among the diagnostic proposition elements, by related factors, populations at risk and associated conditions.

**Table 1:** Background categorization for Ocular dryness, 2022

Antecedent	n	%
Individual factors		
Incomplete eyelid closure (lagophthalmos)	75	41.6
Blinking mechanism decrease	66	36.6
Drugs that damage eye surface homeostasis	66	36.6
Advanced age	55	30.5
Women	53	29.4
Sedation	48	26.6
Interned in the Intensive Care Unit	46	25.5
Mechanical ventilation	45	25.0
Contact lens wearers	40	22.2
Systemic changes (diabetes mellitus, hypertension, hyperthyroidism, chronic kidney failure, multiple organ failure)	34	18.8
Autoimmune diseases (Sjogren’s syndrome, rheumatoid arthritis, systemic lupus erythematosus)	25	13.8
Eye surgical procedures (refractive surgery, cataract surgery, blepharoplasty)	24	13.3
Damage to the ocular surface	24	13.3
Decreased score on the Glasgow coma scale/Decreased level of consciousness	20	11.1
Exposure to digital screens	18	10.0
Vitamin A deficiency	17	9.4
Allergy	08	4.4
Smoking	08	4.4
Exophthalmos	07	3.8
Undergoing procedures at the Surgical Center	06	3.3
Undergoing Hematopoietic Stem Cell Transplantation	06	3.3
Oxygen therapy	06	3.3
Eyelid edema	05	2.7
Undergoing radiotherapy	04	2.2
Impaired corneal reflex	04	2.2
Extended reading	03	1.6
Poorly adapted non-invasive mechanical ventilation masks	02	1.1
Absence of response to cranial nerve pairs reflexes III, IV and VI	01	0.5
Alteration of leukocytes	01	0.5
Environmental factors		
Low humidity	17	9.4
Excessive draft	10	5.5
Use of air conditioning	09	5.0

Twelve consequences were identified, which were subdivided into signs and symptoms and represented the defining characteristics of the proposed diagnosis, presented in Table 2 below.

**Table 2:** Categorization of consequences for Ocular dryness, 2022

Consequences	n	%
Signals		
Conjunctival hyperemia	36	20.0
Decreased tear volume	34	18.8
Excess mucus secretion/excess of ciliary crusts	19	10.5
Chemosis	19	10.5
Dilated blood vessels on the ocular surface	04	2.2
Mucoid filaments	04	2.2
Mucous plaques	04	2.2
Symptoms		
Blurred vision	33	18.3
Burning	29	16.1
Foreign body sensation	25	13.8
Itching	17	9.4
Eye fatigue	16	8.8

The nursing diagnosis constructed and proposed from the concept analysis is structurally described in Table 3 (in English) and in Table 4 (in Portuguese), proposed as a member of domain 11, Security/protection and in Class 2, Physical injury.

**Table 3:** Proposition of the structure of nursing diagnosis Ocular dryness based on the concept analysis, 2022

Domain 11. **Security/Protection** Class 2. **Physical injury** **Ocular dryness**	
**Definition**	
Quantitative insufficiency of the tear film, which may damage the maintenance of ocular surface integrity.	
**Defining characteristics**	
Signs:	Symptoms:
**■** Conjunctival hyperemia **■** Decreased tear volume **■** Excess mucus secretion/excess of ciliary crusts **■** Chemosis **■** Dilated blood vessels on the ocular surface **■** Mucoid filament **■** Mucoid plaque	**■** Blurred vision **■** Burning **■** Foreign body sensation **■** Itching **■** Eye fatigue
**Related factors**	
Individual Factors:	
**■** Incomplete eyelid closure (Lagophthalmos) **■** Blinking mechanism decrease **■** Exposure to digital screens **■** Smoking **■** Exophthalmos	**■** Eyelid edema **■** Impaired corneal reflex **■** Extended reading **■** Absence of response to cranial nerve pairs reflexes III, IV and VI
Environmental factors:	
**■** Low humidity **■** Excessive draft **■** Use of air conditioning	
**At-risk population**	
**■** Advanced age **■** Women **■** Interned in the Intensive Care Unit **■** Contact lens wearers	**■** Undergoing procedures at the Surgical Center **■** Undergoing Hematopoietic Stem Cell Transplantation **■** Undergoing radiotherapy
**Associated conditions**	
**■** Drugs that damage eye surface homeostasis **■** Sedation **■** Mechanical ventilation **■** Systemic changes (diabetes mellitus, hypertension, hyperthyroidism, chronic kidney failure, multiple organ failure) **■** Autoimmune diseases (Sjogren’s syndrome, rheumatoid arthritis, systemic lupus erythematosus) **■** Eye surgical procedures (refractive surgery, cataract surgery, blepharoplasty)	**■** Damage to the ocular surface **■** Decreased score on the Glasgow coma scale/Decreased level of consciousness **■** Vitamin A deficiency **■** Allergy **■** Oxygen therapy **■** Poorly adapted non-invasive mechanical ventilation masks **■** Alteration of leukocytes
**Suggested NOC outcomes**	**Suggested NIC interventions**
Outcomes to Measure Diagnostic Resolution Dry Eye Severity (2110) Outcomes Additional for Measuring Defining Characteristics Dry Eye Severity (2110) Outcomes Associated with Related Factors or Outcomes Intermediate Risk Control: Dry Eye (1927) Smoking Cessation Behavior (1625) Risk Control (1902) Risk Control: Visual Impairment (1916) Risk Control: Sun Exposure (1925) Physical Aging (0113) Neurological Status: Consciousness (0912) Neurological Status: Cranial Sensory/ Motor Function (0913) Surgical Recovery: Immediate Post-Operative (2305) Allergic Response: Localized (0705) Mechanical Ventilation Response: Adult (0411) Medication Response (2301) Immune Hypersensitivity Response (0707)	Suggested Nursing Interventions for Problem Resolution Dry Eye Prevention (1350) Eye Care (1650) Environmental Management: Comfort (6482) Smoking Cessation Assistance (4490) Additional Optional Interventions Medication Administration: Eye (2310) Medication Management (2380) Management Allergy (6410)

**Table 4:** Proposition of the structure of nursing diagnosis Ocular dryness based on the concept analysis (Portuguese translation), 2022

*Domínio 11. **Segurança/Proteção** Classe 2. **Lesão física*** **Ressecamento ocular**	
***Definição***	
*Insuficiência quantitativa do filme lacrimal, que pode comprometer a manutenção da integridade da superfície ocular.*	
***Características definidoras***	
*Sinais:*	*Sintomas:*
**■** *Hiperemia conjuntival* **■** *Volumetria lacrimal diminuída* **■** *Excesso de secreção mucosa/ excesso de crostas ciliares* **■** *Quemose* **■** *Vasos sanguíneos dilatados na superfície ocular* **■** *Filamentos mucoides* **■** *Placas mucosas*	**■** *Visão turva* **■** *Queimação* **■** *Sensação de corpo estranho* **■** *Prurido* **■** *Fadiga ocular*
***Fatores relacionados***	
*Fatores do indivíduo:*	
**■** *Fechamento palpebral incompleto (lagoftalmia)* **■** *Mecanismo de piscar diminuído* **■** *Exposição às telas digitais* **■** *Tabagismo* **■** *Exoftalmia*	**■** *Edema palpebral* **■** *Reflexo corneano prejudicado* **■** *Leitura prolongada* **■** *Ausência de resposta reflexa dos nervos cranianos III, IV e VI*
*Fatores ambientais:*	
**■** *Umidade baixa* **■** *Vento excessivo* **■** *Uso de ar-condicionado*	
***Populações em risco***	
**■** *Idade avançada* **■** *Sexo feminino* **■** *Internados em Unidade de Terapia Intensiva* **■** *Usuários de lentes de contato*	**■** *Submetidos a procedimentos no centro cirúrgico* **■** *Submetidos ao transplante de células-tronco Hematopoiéticas* **■** *Submetidos à radioterapia*
***Condições associadas***	
**■** *Medicamentos que alteram a homeostase da superfície ocular* **■** *Sedação* **■** *Ventilação mecânica* **■** *Alterações sistêmicas (diabetes mellitus, hipertensão, hipertireoidismo, insuficiência renal crônica, falência de múltiplos órgãos)* **■** *Doenças autoimunes (Síndrome de Sjogren, artrite reumatoide, lúpus eritematoso sistêmico)* **■** *Procedimentos cirúrgicos oculares (cirurgia refrativa, cirurgia de catarata, blefaroplastia)*	**■** *Dano à superfície ocular* **■** *Diminuição do escore da escala de coma de Glasgow/redução do nível de consciência* **■** *Deficiência de vitamina A* **■** *Alergia* **■** *Oxigenioterapia* **■** *Máscaras de ventilação mecânica não-invasiva mal adaptadas* **■** *Alteração dos leucócitos*
***Resultados NOC sugeridos***	***Intervenções NIC sugeridas***
*Resultados para Mensurar a Resolução do Diagnóstico* *Gravidade do Olho Seco (2110)* *Resultados Adicionais para Mensurar as Características Definidoras* *Gravidade do Olho Seco (2110)* *Resultados Associados aos Fatores Relacionados ou Resultados Intermediários* *Controle de Risco: Olho Seco (1927)* *Comportamento de Cessação do Tabagismo (1625)* *Controle de Riscos (1902)* *Controle de Riscos: Deficiência Visual (1916)* *Controle de Riscos: Exposição ao Sol (1925)* *Envelhecimento Físico (0113)* *Estado Neurológico: Consciência (0912)* *Estado Neurológico: Função Sensório/Motora Espinal (0913)* *Recuperação Cirúrgica: Pós-Operatório Imediato (2305)* *Resposta Alérgica: Localizada (0705)* *Resposta à Ventilação Mecânica: Adulto (0411)* *Resposta ao Medicamento (2301)* *Resposta Imune de Hipersensibilidade (0707)*	*Intervenções de Enfermagem Sugeridas para Resolução do Problema* *Prevenção contra Ressecamento Ocular (1350)* *Cuidado Ocular (1650)* *Controle do Ambiente: Conforto (6482)* *Assistência para Parar de Fumar (4490)* *Intervenções Opcionais Adicionais* *Administração de Medicamentos: Oftálmica (2310)* *Controle de Medicamentos (2380)* *Controle de Alergias (6410)*

The diagnosis construction started from the multiaxial system of axes described in NANDA-I, since it brings together Axis 1 – diagnostic focus (Ocular dryness) explicit in the title, Axis 2 – diagnostic subject (individual), Axis 3 – judgment (resected), Axis 4 – location (eye), Axis 5 – age (adults) and Axis 7- Category of the diagnosis (focusing on the problem) implicit in the title. In this case, Axis 6 (time) was not relevant for constructing the diagnosis under study.

A definition for the diagnostic label was proposed, in addition to 12 defining characteristics, 12 related factors, seven populations at risk and 13 associated conditions. Moreover, NOC outcomes and NIC interventions were suggested.

To ratify the analyzed data, a fictitious model case was established that exemplified the occurrence of the concept of ocular dryness, described below: Mrs. Ana, 65 years old, smoker, on the 4th day of hospitalization in the ICU due to pneumonia with complications. Subjected to invasive mechanical ventilation, sedated and using antibiotic therapy. During the ocular evaluation, the presence of conjunctival hyperemia and chemosis was observed. When performing the Schirmer I test, a result of seven millimeters was obtained, confirming the decreased tear volume.

The opposite case, established to describe an example of the non-occurrence of the concept, was reported below: Mr João, 57 years old, on the 1st day of admission to the ICU in the postoperative period of hip arthroplasty. It has no associated comorbidities. He is conscious, oriented, on ambient oxygen. In use of analgesics and prophylactic antibiotic therapy. During the ocular evaluation, no changes were found on the surface of the eye. When performing the Schirmer I test, a result of 20 millimeters was obtained, considering adequate tear production.

## DISCUSSION

A unique set of knowledge is a basic characteristic for a profession, since it is essential to know the key concepts or the focus of nursing diagnoses in order to accurately diagnose. Although nurses are able to intervene for the prevention/treatment of dry eyes, the assessment of this human response may not be evident due to the lack of a standardized nursing diagnosis^(^ 8 ^)^.

The structure of the NANDA-I diagnoses helps nurses’ clinical reasoning during care^(^ 11 ^)^. I n view of this, in this study, a diagnostic proposition was constructed focusing on the problem of ocular dryness, with the aim of filling the existing gap in the NANDA-I classification^(^ 8 ^)^.

A total of 180 published studies were used, identified from the scoping review, for the concept analysis, in order to identify the attributes, antecedents and consequences of ocular dryness in adult patients admitted to the ICU.

Regarding the year of publication of the studies identified in the scoping review, it was observed that most articles were published in the last five years, with levels of evidence with a strong degree of recommendation, in order to represent a recent and appropriate discussion of the subject. This result may be related to the greater interest in studies related to ocular health, with emphasis on dry eye/ ocular dryness in recent years, a care that is sometimes neglected, especially in critical care settings, such as the ICU^(^ 1,6,7,8,9,10,11,12,13,14,15,16,17,18,19 ^)^.

Two attributes were identified to conceptually define the diagnosis Ocular dryness, such as tear film deficiency and ocular signs and/or symptoms. The tear film is highly stable, its layers are cohesive during eye movements and its purpose is to lubricate and maintain ocular structures. When there is a decrease in tear volume, structure maintenance and lubrication is compromised^(^ 20 ^)^.

The tear film is important for maintaining the ocular surface’s health and function by minimizing friction and dryness on the eye surface as well as being important for cornea oxygenation and protection against infection^(^ 21 ^)^. The concept of tear deficiency was first proposed in 1903 by Schirmer, who developed the Schirmer test, capable of detecting aqueous deficiency in tears^(^ 22 ^)^.

In this regard, tear film deficiency is understood as a reduction in tear production and/or increased evaporation due to changes in the responsible ocular mechanisms that cause damage to the lacrimal gland and cause a decrease in tear production or exposure of the ocular surface with consequent elevation from tear evaporation^(^ 20,22,23 ^)^.

The initial stage of tear film deficiency/insufficiency can be exacerbated and promote the appearance of ocular signs and/ or symptoms, such as hyperemia, quantitative reduction of tear volume, mucous secretion, chemosis, dilated blood vessels, mucoid filaments, mucous plaques, blurred vision, burning, foreign body sensation, pruritus and fatigue, capable of altering the ocular surface integrity maintenance and generating potential harm to health^(^ 6,23,24 ^)^.

From the above, ocular dryness can be understood as an initial condition of tear film deficiency that alters the amount of tears. Therefore, it can behave as an isolated human response under the responsibility of nursing or a response associated with particular stages of ocular diseases, such as dry eye, keratitis and corneal injury. Thus, the identification of attributes was fundamental for the conceptual definition of the proposed diagnosis.

Defining characteristics for the diagnostic proposition were identified, and it is observed that they can be divided into signs and symptoms. The most frequent were conjunctival hyperemia, decreased tear volume, blurred vision, burning, foreign body sensation and mucous secretion/excess ciliary crusts.

Corroborating these findings, another study describes that hyperemia and mucous secretion are significant clinical signs for the evaluation of patients admitted to the ICU, as they predict ocular dryness^(^ 6 ^)^. Another study also revealed a high presence of conjunctival hyperemia (56.2%) in the assessed patients^(^ 25 ^)^. Still, authors described that burning sensation in the eyes, ocular fatigue, blurred vision, ocular itching, sandy sensation and presence of foreign body occur due to the mechanical interaction of the palpebral conjunctiva and the surface of the resected cornea^(^ 26 ^)^.

Community-based research identified the most commonly reported symptoms in patients with ocular surface disorders, such as a gritty sensation (53.4%), a burning/stinging sensation (48.3%) and a feeling of dryness (35.6%)^(^ 27 ^)^.

The related factors identified were classified into individual and environmental factors. Regarding individual factors, incomplete eyelid closure (lagophthalmos) and blinking mechanism decrease stood out.

Lagophthalmos occurs due to suppression of the orbicularis muscle function by physiological mechanisms during sleep or secondary to drug treatment. Such exposure may result in increased tear film evaporation and dryness of the ocular surface^(^ 28 ^)^. Study points out in its results the exposure of the eyeball as the main risk factor for alterations of the ocular surface^(^ 1 ^)^.

Research conducted in the ICU described a significant relationship between the presence of lagophthalmos and the development of exposure keratopathy^(^ 29 ^)^. Therefore, the greater the degree of exposure, the greater the impairment of the cornea and, therefore, the cornea becomes susceptible to the undesirable effects of tear evaporation and tear film instability^(^ 6,30 ^)^.

The blinking mechanism within the parameters of normality plays an integral role in the ocular surface and in the homeostasis of the tear film, since this condition of decrease represents the limitation of the spontaneous movement of the blinking reflex (less than or equal to five times per minute), which compromises tear drainage pumping, microorganism removal and uniform tear distribution, in addition to favoring increased tear film evaporation by environmental factors^(^ 9,31,32 ^)^.

According to the related environmental factors identified, the use of an air conditioner, together with low humidity and excessive draft (excessive ventilation), is considered a condition of the external environment that exposes the ocular surface to the risk of dryness. Low ambient humidity (<30%) can be caused by the use of an air conditioner and direct facial exposure to excessive air flows. The use of poorly adapted macronebulizers, Venturi masks, nasal catheters and non-invasive mechanical ventilation or oxygen therapy devices increases tear evaporation and, when associated with reduced tear production, increases the palpebral fissure and decreases the blinking mechanism^(^ 7,10,33 ^)^.

According to the populations at risk represented in the literature for diagnostic proposition Ocular dryness, those with advanced age and females were the most commonly found. Aging is a state that comprises the loss of androgens, resulting in changes in the main lacrimal gland and an increase in the rate of evaporation due to the modification of the lipid layer produced by the meibomian glands^(^ 10,34 ^)^. With regard to the female sex, women produce androgens in smaller quantities than men, with a greater possibility of compromising the function of the lacrimal glands^(^ 7,10,34 ^)^.

Patients outside the ICU environment described that individuals aged 60 years or older were about three times more likely to have ocular dryness than those aged between 40 and 59 years^(^ 27 ^)^. Other factors related to decreased tear volume in elderly patients are the increased prevalence of systemic diseases in the more advanced age group, such as diabetes, rheumatoid arthritis as well as those with the use of systemic and topical medications that interfere with normal tear function^(^ 35 ^)^.

Among the identified associated conditions, drugs that alter homeostasis of the ocular surface, the use of sedation and mechanical ventilation deserve to be highlighted, as they are presented in an expressive way in the sample studies.

These drugs were mainly those that act on cholinergic receptors, which cause a reduction or blockade of acetylcholine activity, with a decrease in the tonic contraction of the orbicularis muscle, resulting in incomplete eyelid closure, corneal exposure and dryness. Other medications are those that alter tear film formation and decrease aqueous tear layer production, with potential damage to the ocular surface due to dryness^(^ 10,26,29,31,36 ^)^.

Sedatives and neuromuscular blockers are also related to ocular dryness, because they inhibit the orbicularis oculi muscle contraction with consequent incomplete closure of the eyelid, eliminating the blinking reflex, one of the main eye protection mechanisms. Thus, the presence of ocular surface disease is closely related to the degree of lagophthalmos, which, in turn, was closely related to depth of sedation^(^ 25 ^)^.

Mechanical ventilation comprises invasive or non-invasive mechanical ventilatory support with positive end-expiratory pressure (PEEP). The use of high PEEP can compromise the eye, by increasing intrathoracic pressure and producing effects that potentiate venous stasis, facial edema and decrease ocular perfusion^(^ 10,29,31,32 ^)^.

Considered as a common treatment in critically ill patients, the use of invasive mechanical ventilation generally requires sedatives to better adjust ventilatory parameters and, in some situations, neuromuscular blockers, with the potential to affect natural eye protection mechanisms and increase the risk of ocular surface disorders^(^ 10,28 ^)^.

Although there are some barriers to providing eye care in the ICU, such as lack of time, shortage of trained manpower, and lack of adequate knowledge and skills, nurses can play an important role in establishing an early diagnosis and ocular dryness during care delivery. Including a routine eye assessment can prevent unnoticed ocular surface problems, as well as the later complications that are likely to occur, and thereby ensure an increased quality of life for patients^(^ 29,37,38,39 ^)^.

### Study limitations

This study has limitations that may be related to the data sources selected for the identification of studies as well as the limit of four languages, which may have collaborated to hide other relevant research on the topic addressed.

### Contributions to nursing

The implementation of studies aimed at continuous development and the use of nursing classification systems help advance knowledge and nursing efforts to gain greater visibility in the health policy scenario by using their classification systems as an object. Thus, the execution of this research promotes the collection of scientific evidence that underlies the nursing diagnostic proposal Ocular dryness, providing subsidies for nursing practice, in the sense of early detection and prevention of ocular dryness and other subsequent injuries in the ICU, with targeted knowledge focused on this human response.

## CONCLUSIONS

This study allowed us to identify, through a concept analysis, two attributes, 32 antecedents and 12 consequences of the diagnostic focus of the research. Based on this, a proposal for nursing diagnosis Ocular dryness was constructed for the NANDA-I taxonomy. The formulated nursing diagnosis was proposed as part of domain 11, Safety/protection, in class 2, Physical injury, with 12 defining characteristics, 12 related factors, seven populations at risk and 13 associated conditions.

The new problem-focused nursing diagnostic proposal, based on the NANDA-I level of evidence 2.1.1, may provide more targeted care in the sense of promoting early detection of the problem and carrying out interventions that reduce the risk of ocular damage, by supporting nurses’ clinical reasoning for diagnostic accuracy.
